# Metal–Organic Frameworks as Promising Photosensitizers for Photoelectrochemical Water Splitting

**DOI:** 10.1002/advs.201500243

**Published:** 2015-11-19

**Authors:** Liping Zhang, Ping Cui, Hongbin Yang, Jiazang Chen, Fangxing Xiao, Yuanyuan Guo, Ye Liu, Weina Zhang, Fengwei Huo, Bin Liu

**Affiliations:** ^1^ Energy Research Institute@NTUInterdisciplinary Graduate SchoolNanyang Technological UniversitySingapore637141Singapore; ^2^ Division of Chemical and Biomolecular EngineeringSchool of Chemical and Biomedical EngineeringNanyang Technological University62 Nanyang DriveSingapore637459Singapore; ^3^ School of Materials Science and EngineeringNanyang Technological University50 Nanyang AvenueSingapore639798Singapore; ^4^ Key Laboratory of Flexible Electronics (KLOFE) and Institute of Advanced Materials (IAM)Jiangsu National Synergistic Innovation Center for Advanced Materials (SICAM)Nanjing Tech University (NanjingTech)30 South Puzhu RoadNanjing211816P.R. China

**Keywords:** metal–organic frameworks, photosensitizer, TiO_2_, photoelectrochemical water splitting

## Abstract

**Ti‐based metal‐organic frameworks (MOFs) are demonstrated as promising photosensitizers** for photoelectrochemical (PEC) water splitting. Photocurrents of TiO_2_ nano wire photoelectrodes can be improved under visible light through sensitization with aminated Ti‐based MOFs. As a host, other sensitizers or catalysts such as Au nanoparticles can be incorporated into the MOF layer thus further improving the PEC water splitting efficiency.

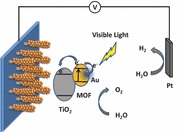

## 1 Introduction

Since the first discovery of photoelectrochemical (PEC) water splitting by Fujishima and Honda in the 1970s,^1^ artificial photosynthesis that converts solar energy into chemical fuels—mimicking what nature does—has received great attention in the scientific community. Among the various studied materials, titanium dioxide (TiO_2_) has attracted the greatest attention due to its nontoxicity, physical and chemical stability, and peculiar electronic and optical properties.^2^ However the wide band gap of TiO_2_ limits its absorption of visible light, which restricts the solar energy conversion efficiency. Over the past few years, considerable efforts have been made to tune the photoresponse of TiO_2_ to improve the visible light absorption, such as metallic (e.g., Fe, Zr) or non‐metallic (e.g., N, F, C, S, B) doping,^3^, ^4^, ^5^, ^6^, ^7^, ^8^, ^9^ and dye molecule^10^, ^11^ or narrow band gap semiconductor (e.g., CdS, CdSe) sensitization.^12^, ^13^, ^14^, ^15^, ^16^ Among which, sensitization of TiO_2_ appears to be a promising strategy for harvesting solar energy by enhancing visible light absorption and improving charge separation.^17^, ^18^ However, dye sensitizers usually have low light absorption coefficient and are effective only when they are chemically bound onto the surface of the semiconductor, with performance depending on the surface area as well as structure of the semiconductor.^19^, ^20^ Furthermore, dye molecules are generally catalytically inactive, which have to be combined with molecular catalysts to realize noticeable photocatalytic activities. Alternatively, narrow band gap semiconductors with large light absorption coefficient are frequently used as photosensitizers for TiO_2_ in solar energy conversion. However the stability and toxicity for most of the narrow band gap semiconductors remain a serious problem.

In this work, we demonstrate a new type of photosensitizers, for PEC water splitting: metal–organic frameworks (MOFs). MOFs as a new class of hybrid materials possess highly ordered framework structure and large surface area. Due to their fascinating chemical and physical properties, MOFs are expected to be useful in a large scope of applications, including gas storage^21^, ^22^ and separation,^23^ chemical sensing,^24^ biomedicine^25^ and heterogeneous catalysis.^26^, ^27^ More recently, MOFs are emerging as a new type of photocatalyst.^28^ Having combined metal nodes and bridging ligands, MOFs provide the possibilities of periodical arrangement of light harvesting and catalytic components in a single structure, just like the natural photosynthetic system, making them promising candidates for solar energy conversion. Among different types of MOFs, Ti‐based MOFs are expected with good photocatalytic properties due to the presence of high density of Ti‐oxo clusters.^29^, ^30^ Under light irradiation, electrons are photogenerated in organic ligands and then transferred to Ti‐oxo clusters.^30^ Based on a recent study,^31^ MIL‐125 (a Ti‐based MOF) has a more negative LUMO (lowest unoccupied molecular orbital) level than the conduction band edge of TiO_2_, making electron injection from MIL‐125 to TiO_2_ thermodynamically feasible. Thus herein, we studied a series of Ti‐based MOFs as photosensitizers on TiO_2_ photoanode for PEC water splitting. Our results showed that the PEC water oxidation performance of TiO_2_ could be significantly enhanced by applying MOFs as photosensitizers under visible light illumination.

This is an open access article under the terms of the Creative Commons Attribution License, which permits use, distribution and reproduction in any medium, provided the original work is properly cited.

## 2 Results and Discussion

**Figure 1:**
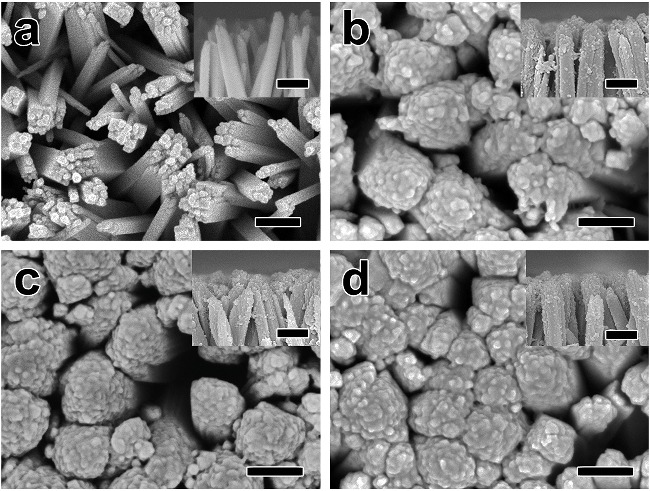
FESEM images of a) TiO_2_ nanowire arrays, b) TiO_2_/MIL‐125 nanowire arrays, c) TiO_2_/MIL‐125(NH_2_) nanowire arrays, and d) TiO_2_/MIL‐125(NH_2_)_1,2_ nanowire arrays. Scale bars: 200 nm.

Three Ti‐based MOFs (MIL‐125, MIL‐125(NH_2_), MIL‐125(NH_2_)_1,2_) were studied as photosensitizers for PEC water splitting. The TiO_2_/MOF hybrid nanocomposite was prepared on an FTO substrate by a two‐step solution phase method. In the first step, vertically oriented, single crystalline TiO_2_ nanowire arrays were grown on FTO substrate based on a hydrothermal method. The as‐grown ordered one‐dimensional TiO_2_ nanostructures possess large surface area as well as short minority carrier diffusion pathways (perpendicular to the nanowire growth direction), which could serve as excellent photoanode for PEC water splitting.^32^, ^33^ **Figure** **1**a and Figure S1 (Supporting Information) show both the top view and cross‐sectional view FESEM images of the TiO_2_ nanowire arrays, from which the average diameter and length of the TiO_2_ nanowires were determined to be around 100 nm and 1.5 μm, respectively.

**Figure 2:**
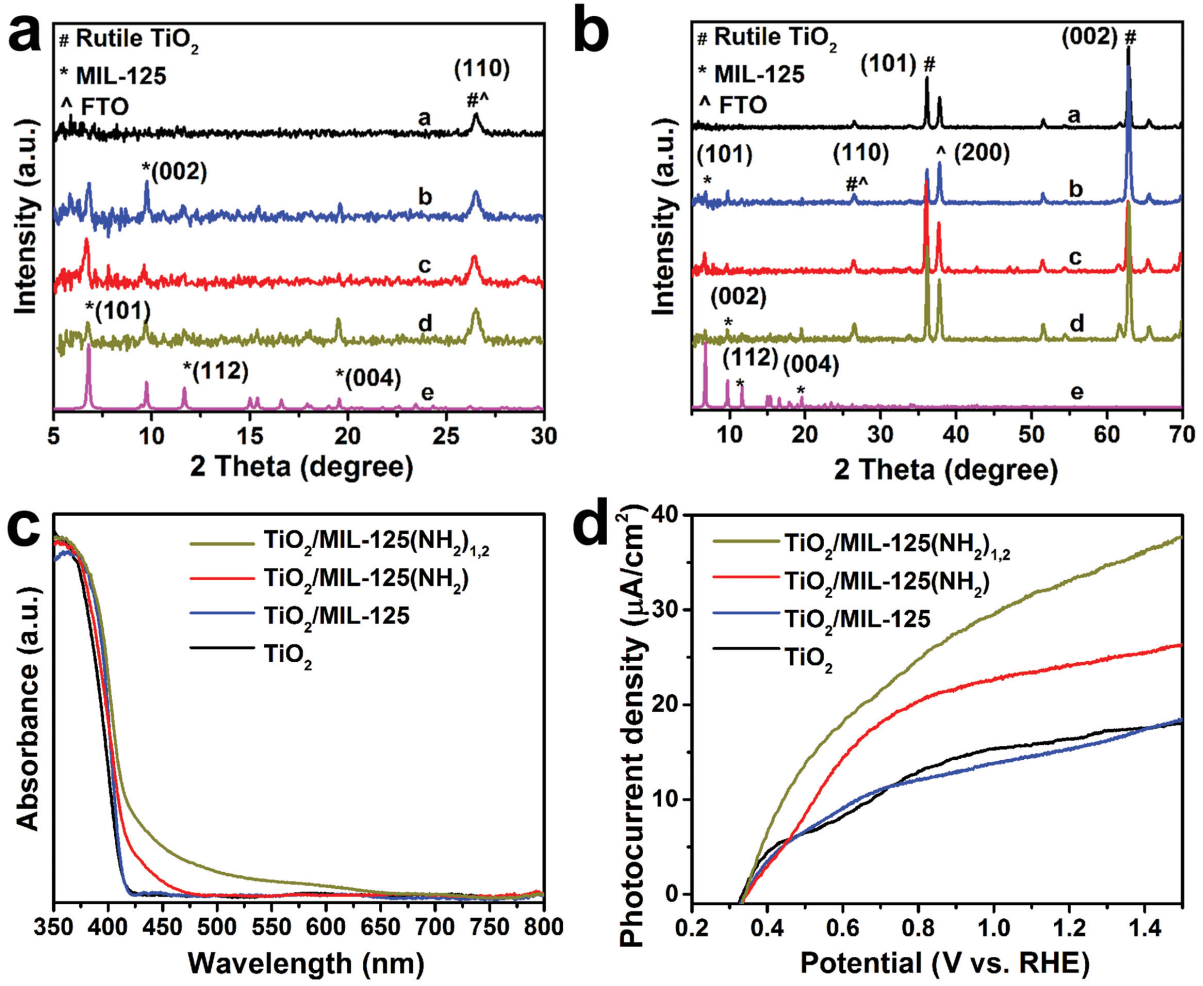
a,b) XRD patterns of TiO_2_ and TiO_2_/MOF nanocomposites: curve a) TiO_2_, b) TiO_2_/MIL‐125, c) TiO_2_/MIL‐125(NH_2_), d) TiO_2_/MIL‐125(NH_2_)_1,2_ and e) simulated MIL‐125). c) UV–vis diffuse reflectance spectra of TiO_2_ and TiO_2_/MOF nanocomposites. d) J–V curves of TiO_2_ and TiO_2_/MOF nanocomposites.

Subsequently, in the second step, Ti‐based MOFs were grown on the surface of TiO_2_ nanowires to form a core‐shell hybrid nanostructure. In this step, it was found that modification of the TiO_2_ surface was critical in promoting the heterogeneous nucleation and growth of MOF nanostructures. Without surface modification, the Ti‐based MOF grew homogeneously in solution, resulting in phase segregated TiO_2_ and MOF nanostructures (Figure S2, Supporting Information). The ligand (BDC or BDC‐NH_2_) used to modify TiO_2_ has –COOH functional groups, which bind strongly with the surface Ti atom on TiO_2_ and thus create nuclei sites for the MOF growth. Figure 1b–d show FESEM images of TiO_2_ nanowire arrays coated with MIL‐125, MIL‐125(NH_2_) and MIL‐125(NH_2_)_1,2_, respectively. It can be observed that the surface of the nanowires becomes rougher after coating with MOF nanostructures, accompanied by an increase in nanowire diameter, indicating successful coating of MOF on the surface of TiO_2_ nanowires. The XRD patterns as displayed in **Figure** **2**a,b also show both diffractions from TiO_2_ and MOFs, which match perfectly with the FESEM observation.

Depending on the choices of ligands, the Ti‐based MOFs show different light absorption properties. Figure 2c shows the UV–vis diffuse reflectance spectra of TiO_2_ and TiO_2_/MOF nanocomposites. Both TiO_2_ and TiO_2_/MIL‐125 show similar light absorption onset, which is around 420 nm, suggesting the wide band gap nature for both TiO_2_ and MIL‐125. Based on the literature,^34^ the HOMO (highest occupied molecular orbital)–LUMO gap of MIL‐125 is 3.8 eV; i.e., MIL‐125 can only absorb light in the UV spectrum. To reduce the HOMO–LUMO gap, amine modified ligands were used for growing MIL‐125. Amines are strong electron‐donating substituents, which could help to raise the HOMO level of MOFs while maintaining the LUMO level, thus reducing the HOMO–LUMO gaps. After amine modification, monoaminated MIL‐125(NH_2_) shows an obvious red‐shift in light absorption towards visible light, while partially diaminated MIL‐125(NH_2_)_1,2_ possesses an even narrower HOMO–LUMO gap with absorption onset at around 650 nm, which correspond well with the yellow and dark brown colors of MIL‐125(NH_2_) and MIL‐125(NH_2_)_1,2_ (Figure S3, Supporting Information). Clearly, ligand modification offers an effective way to tune the HOMO–LUMO gaps of MOFs, thus changing the optical properties.

Photocatalytic properties of as‐prepared TiO_2_/MOF nanocomposites were evaluated by performing PEC water splitting. Both TiO_2_ and TiO_2_/MOF samples with fixed sample area were used as the photoanode. Figure 2d displays the J–V curves for water splitting under AM1.5 illumination with UV light cutoff (λ > 420 nm). It is clear to see that the pristine TiO_2_ nanowire electrode has a very small photocurrent density of only 10 μA cm^−2^ at 0.75 V vs. RHE (reversible hydrogen electrode), due to the large band gap of rutile TiO_2_ (3.0 eV). When sensitized with Ti‐based MOFs, different PEC performances were realized. MIL‐125 sensitized TiO_2_ showed a comparable PEC performance as the pristine TiO_2_ electrode, which is due to the fact that MIL‐125 can only absorb UV light. On the contrary, significant improvements in photocurrent were observed in both MIL‐125(NH_2_) and MIL‐125(NH_2_)_1,2_ sensitized TiO_2_ electrodes, resulting from much enhanced visible light absorption in these aminated MOFs. The LUMO level of MIL‐125 is more negative than the conduction band edge of TiO_2_,^31^ as a result, electron transfer from MIL‐125 to TiO_2_ will proceed favorably. As ligand modification merely affects the LUMO level of MOFs,^34^ it is anticipated that the electron transfer from MIL‐125(NH_2_) or MIL‐125(NH_2_)_1,2_ to TiO_2_ will be unaffected. Upon visible light illumination, electrons in the HOMO level of MIL‐125(NH_2_) or MIL‐125(NH_2_)_1,2_ are photoexcited into the LUMO level, which will be quickly injected into the conduction band of TiO_2_ and then be transported through TiO_2_ to reach the counter electrode to reduce water. Simultaneously, the holes in the HOMO level of MOFs will drive water oxidation to evolve oxygen. Thus, the overall PEC performance will be affected by the light absorption as well as rate of chemical reactions (water reduction and oxidation).

Increasing the MOF layer thickness could increase the photocurrent density because of enhanced light absorption; however, if the MOF layer becomes too thick, the poor electron transport in the MOF layer could lead to significant charge recombination and thus decrease the photocurrent density. To further improve light absorption while at the same time keeping a thin MOF layer to enhance water oxidation kinetics, Au nanoparticles were specifically selected to modify MOF sensitized TiO_2_ photoelectrode. Au nanoparticles not only have strong light absorption in the visible solar spectrum due to localized surface plasmon resonance (LSPR), which can be utilized to increase the light absorption cross‐section of the photosensitizer, but also have the capability to catalyze water oxidation reaction. MIL‐125(NH_2_) sensitized TiO_2_ was further decorated with Au nanoparticles. Figures S4–S6 (Supporting Information) show the XRD pattern, XPS spectra and TEM images of Au nanoparticles modified TiO_2_/MIL‐125(NH_2_) nanocomposite. In the wide scan XPS spectra (Figure S5a), the newly appeared N 1s peak in TiO_2_/MIL‐125(NH_2_) and TiO_2_/MIL‐125(NH_2_)/Au suggest the successful coating of MOF on the TiO_2_ photoanode. While the Au 4f peaks in TiO_2_/MIL‐125(NH_2_)/Au reveal the successful decoration of Au nanoparticles on MOF coated TiO_2_ photoanode. After MOF coating, the O 1s peak at 533 eV can be assigned to the –COO^−^ functional groups from MOF ligand (Figure S5b–d). The chemical states of neither O nor N were changed after Au nanoparticles were further loaded (Figure S5b–f). From TEM images (Figure S6a,b), plenty of Au nanoparticles are clearly observed. The Au nanoparticles are uniformly decorated on the TiO_2_/MIL‐125(NH_2_) nanocomposite as confirmed by the STEM‐EDX mapping measurement (Figure S7a–e, Supporting Information). After loading Au NPs, an additional light absorption peak centered at 550 nm resulting from the Au SPR excitation further confirms the successful decoration of Au NPs on TiO_2_/MIL‐125(NH_2_) (Figure S7f).

As electrochemical processes are interfacial, the surface area of the photoelectrode is crucial in our PEC water splitting system. The electrode surface area can be estimated by measuring the double‐layer capacitance,^35^ which was done by cycling the electrode over a narrow potential window (0–0.1 V vs. Ag/AgCl) over which no Faradaic processes took place. The CVs were recorded over a range of scan rates between 1–100 mV sec^−1^ and the differences between the anodic and cathodic plateau currents were plotted as a function of the scan rate (Figure S8, Supporting Information), which yielded a straight line, intercepting the origin and possessing a slope equal to the double‐layer capacitance of the electrode. From Figure S8a, the TiO_2_/MIL‐125(NH_2_) possesses the largest slope—i.e., largest surface area—resulting from the highly porous structure of MOF materials. After incorporating Au nanoparticles, surface area decreased due to blocking of MOF pores by Au nanoparticles.

**Figure 3:**
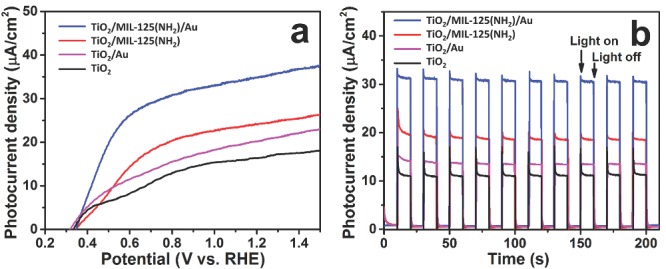
PEC performance of TiO_2_ and sensitized TiO_2_ nanowire arrays under visible light illumination (*λ* > 420 nm). a) *J*–V curves. b) *J*–*t* curves upon chopped illumination at 0.75 V vs. RHE.

PEC water oxidation performance was further enhanced by loading Au nanoparticles onto the TiO_2_/MIL‐125(NH_2_) photoelectrode. The stabilized photocurrent was improved by nearly 50% from 20 μA cm^−2^ to 30 μA cm^−2^ at a bias of 0.75 V vs. RHE under visible light illumination (**Figure** **3**a). As a control experiment, Au nanoparticle modified TiO_2_ was also fabricated by the same procedure with TiO_2_/MIL‐125(NH_2_). The enhancement of photocurrent can be attributed to two factors. Firstly, the plasmon‐induced visible light absorption of Au NPs can increase the light absorption cross‐section of MIL‐125(NH_2_), which in turn increases the light harvesting efficiency. Secondly, upon light illumination, LSPR generates a strong electromagnetic field close to the surface of Au nanoparticles. This electromagnetic field can modify the band structure at the interface between Au and other semiconductor,^36^ which could facilitate charge separation and reduce charge recombination. Besides, the catalytic activity of Au for water oxidation should not be ignored, which also played a role to improve the water oxidation performance.

**Figure 4:**
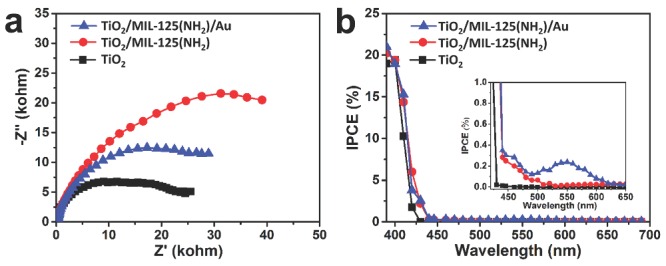
PEC performance of TiO_2_ and sensitized TiO_2_ nanowire arrays under visible light illumination (*λ* > 420 nm). a) Nyquist plots. b) IPCE curves.

To get a deeper understanding on the improved PEC water oxidation performance, electrochemical impedance spectroscopy was carried out to study the charge transfer processes across the electrode/electrolyte interface. **Figure** **4**a shows the electrochemical impedance Nyquist plots. The semicircles in the Nyquist plot provide information on the charge transfer processes.^16^ From Figure 4a, it is clear that TiO_2_/MIL‐125(NH_2_) has a much larger charge transfer resistance at the electrode/electrolyte interface than TiO_2_. This observation should not be surprising as MOFs usually have poor electrocatalytic activities. By decorating Au nanoparticles on TiO_2_/MIL‐125(NH_2_), a reduction in diameter of the semicircle was noticed in the Nyquist plot, indicating improved charge transfer, which could be attributed to the improved charge separation induced by LSPR of Au nanoparticles, as well as enhanced water oxidation kinetics.

The stability of the MOF‐sensitized TiO_2_ photoelectrodes was examined by recording photocurrent as a function of time during chopped and continuous visible light illumination at a bias of 0.75 V vs. RHE as shown in Figure 3b and Figure S9 (Supporting Information), respectively. All photoelectrodes displayed current spikes, which is due to the slow OER kinetics. However, the current spike became smaller after decorating Au nanoparticles, indicating improved OER kinetics, which is in accordance with our electrochemical impedance data as discussed previously. After reaching steady‐state, photocurrent density for TiO_2_, TiO_2_/Au, TiO_2_/MIL‐125(NH_2_) and TiO_2_/MIL‐125(NH_2_)/Au could reach 11 μA cm^−2^, 14 μA cm^−2^, 20 μA cm^−2^, and 32 μA cm^−2^, respectively. Furthermore, these photocurrents are highly repeatable during 10 on–off circles, indicating good photostability of the MOF‐sensitized electrode under strong oxidizing environment.

An incident photon‐to‐current conversion efficiency (IPCE) test was further carried out to examine the current contributions. Figure 3b shows the IPCE spectra of TiO_2_ and MOF‐sensitized TiO_2_ photoelectrodes, which match well with the results of UV–vis absorption (Figure S7f, Supporting Information). In the wavelength region between 420 nm and 500 nm, TiO_2_/MIL‐125(NH_2_) shows an obvious enhancement of IPCE as compared to the pristine TiO_2_, due to improved visible light absorption of MIL‐125(NH_2_), resulting in enhanced overall PEC performance. Loading Au nanoparticles on TiO_2_/MIL‐125(NH_2_) creates an addition IPCE peak centered at 550 nm, arising from the LSPR absorption of Au. It is clear that modification of MIL‐125(NH_2_) and Au nanoparticles on TiO_2_ nanowires significantly improves the visible light absorption and thus enhances the PEC water oxidation performance.

The advantages of using MOFs as photosensitizers are as following: first, the great chemical tailorability enables tuning of the optical and catalytic properties of MOFs at the molecular scale, which can benefit both the light absorption as well as catalytic reactions; second, the highly porous structure of MOFs can also be utilized to incorporate other functional molecules such as photosensitizers or molecular catalysts to work synergistically to improve the photocatalytic efficiency.

## 3 Conclusions

In summary, Ti‐based MOFs have been demonstrated as promising photosensitizers for PEC water splitting. When applied as the photoanode for solar water oxidation, the photocurrent of TiO_2_ nanowire photoelectrode could be improved by nearly 100% under visible light through sensitization with aminated Ti‐based MOFs. By coupling with plasmonic Au nanoparticles, the light absorption efficiency could be further improved due to the LSPR effect. Considering the diversity of MOFs, a large number of MOFs are expected to work as promising photosensitizers or photocatalysts for solar‐driven energy conversion and/or environmental remediation.

## 4 Experimental Section

*Materials*: All chemicals were purchased from Sigma‐Aldrich without further purification. Fluorine‐doped SnO_2_ (FTO) glass was purchased from Latech Scientific Supply.

*Growth of TiO_2_ Nanowire Arrays on FTO*: Oriented rutile TiO_2_ nanowire arrays were grown on FTO substrate using a hydrothermal method based on our previous report.^37^ Briefly, 0.83 mL of tetra‐n‐butyl orthotitanate (TBOT) was mixed with 50 mL of 6 m hydrochloric acid and stirred for 5 min. Afterwards, the growth solution was transferred into a 100 mL Teflon‐lined stainless steel autoclave, followed by placing a few pieces of pre‐cleaned FTO glass. The hydrothermal reaction was conducted in an electronic oven at 150 °C for 5 h. After reaction, the TiO_2_ covered FTO glass was taken out, rinsed thoroughly with deionized water and dried with flowing nitrogen. To improve the crystallinity and conductivity of TiO_2_ nanowire arrays, further annealing was performed in air at 450 °C for 30 min.

*Preparation of TiO_2_/MOF Nanocomposites*: TiO_2_ nanowires were firstly modified with 1,4‐benzenedicarboxylate(BDC) or BDC‐NH_2_, which are the ligands used for growing MIL‐125 and MIL‐125‐NH_2_ respectively. In a particular experiment, 4 pieces of FTO covered by TiO_2_ nanowire arrays were immersed in 4 mL of 20 mm BDC (DMF) or BDC‐NH_2_ (DMF) solution, and kept at 120 °C for 3 h. To grow TiO_2_/MOFs composites, redistilled dimethylformamide (DMF) and methanol (MeOH) were used as the solvent. To 5 mL of DMF/MeOH (v/v = 9/1), 0.3 mmol of BDC and 26 μL of TBOT were firstly mixed in a 20 mL Teflon‐lined autoclave, to which four pieces of FTO covered with TiO_2_ nanowires were placed against the wall of Teflon‐liner with the TiO_2_ side facing down. The solvothermal reaction was conducted at 150 °C for 72 h to grow TiO_2_/MIL‐125. TiO_2_/MIL‐125(NH_2_) and TiO_2_/MIL‐125(NH_2_)_1,2_ were prepared using the same protocol with the same amount of BDC‐NH_2_ ligand and BDC‐(NH_2_)_2_/BDC‐(NH_2_) ligand (molar ratio, 1/9), respectively.

*Preparation of TiO_2_/MIL‐125(NH_2_)/Au Nanocomposite*: HAuCl_4_ was used as gold precursor to prepare Au nanoparticles. Firstly, 2 mm HAuCl_4_ (MeOH) solution was prepared and the pH was adjusted to 7. Subsequently, TiO_2_/MIL‐125(NH_2_) samples preheated at 120 °C for 10 h were soaked into the HAuCl_4_ solution for 3 h, followed by washing with MeOH. After drying, gold precursors were reduced by 0.2 m NaBH_4_ solution for 5 min.

*Characterization*: X‐ray diffraction (XRD) patterns were collected on a Bruker AXS D8 Advance diffractometer using nickel‐filtered Cu Kα radiation (*λ* = 1.5406 Å). Field emission scanning electron microscope (FESEM) images were taken on a JEOL JSM‐7600 with an accelerating voltage of 5 kV. Transmission electron microscopy (TEM) images were taken on a JEOL JEM 2010F at an accelerating voltage of 200 kV. STEM‐EDX elemental mapping was conducted on a JEOL JEM 2100F. UV–visible spectra were recorded using a Perkin‐Elmer Lambda 900 UV–vis–NIR spectrometer equipped with an integrating sphere. X‐ray photoelectron spectroscopy (XPS) measurements were conducted on an ESCALAB 250 photoelectron spectrometer (Thermo Fisher Scientific) at 2.4 × 10^−10^ mbar using a monochromatic Al Ka X‐ray beam (1486.60 eV). Binding energies (BE) were calibrated to the carbon BE of 284.60 eV.

*Photoelectrochemical Measurements*: Photoelectrochemical properties of TiO_2_/MOF nanocomposites were studied on CHI 660D electrochemical workstation in a standard three‐electrode setup with Ag/AgCl (3m NaCl) as the reference electrode and a platinum plate as the counter electrode under simulated sunlight from a 300‐Watt Xenon lamp (Newport, Oriel, 91160) equipped with an AM 1.5G filter (Newport, 81094) and a 420 nm long pass filter (Newport, FSQ‐GG420). The light source was calibrated using a standard Si photodiode. In all cases, a 0.5 m Na_2_SO_4_ solution (pH = 6.5) was used as the electrolyte. All potentials were converted to values relative to RHE using the following equation:

(1) $$E_{\mathrm{RHE}}=E_{\mathrm{Ag/AgCl}}+0.0591\;\;\times \;\;\mathrm{pH}+E_{\mathrm{Ag/AgCl}}^{o}$$

where *E* ^o^_Ag/AgCl_ is the standard potential of Ag/AgCl relative to RHE at 25 °C (0.197 V). The working area of the photoelectrode was fixed at 0.25 cm^2^ for each experiment. The electrochemical impedance spectroscopy measurements were performed by applying a frequency ranging from 10^−2^ to 10^5^ Hz with AC amplitude of 10 mV under visible light illumination (420 nm cut‐off) at open‐circuit voltage condition. The incident photon‐to‐current conversion efficiency (IPCE) was measured at a bias of 0.2 V vs. Ag/AgCl. The monochromatic light was supplied by a 300‐Watt Xe lamp irradiation through a monochromator (Newport). A chopper was placed in front of the monochromator, and the signal was collected using a lock‐in radiometry (Merlin) after amplification by the current preamplifier.

## Supporting information

As a service to our authors and readers, this journal provides supporting information supplied by the authors. Such materials are peer reviewed and may be re‐organized for online delivery, but are not copy‐edited or typeset. Technical support issues arising from supporting information (other than missing files) should be addressed to the authors.

Supplementary
