# Supplementary material for: Metal–Organic Frameworks as Promising Photosensitizers for Photoelectrochemical Water Splitting
Source: Adv Sci (Weinh). 2015 Nov 19;3(1):1500243. doi: 10.1002/advs.201500243 (PMC5063145; doi:10.1002/advs.201500243)
Supplement: Supplementary file 1 — Supplementary [file ADVS-3-0p-s001.pdf]

Copyright WILEY-VCH Verlag GmbH & Co. KGaA, 69469 Weinheim, Germany, 2015.

ADVANCED SCIENCE
Open Access

Supporting Information

for *Adv. Sci.*, DOI: 10.1002/advs. 201500243

Metal–Organic Frameworks as Promising Photosensitizers for Photoelectrochemical Water Splitting

Liping Zhang, Ping Cui, Hongbin Yang, Jiazang Chen, Fangxing Xiao, Yuanyuan Guo, Ye Liu, Weina Zhang, Fengwei Huo, and Bin Liu**

Supporting Information

Metal-Organic Frameworks (MOFs) as Promising Photosensitizers for Photoelectrochemical Water Splitting

Liping Zhang, Ping Cui, Hongbin Yang, Jiazang Chen, Yuanyuan Guo, Ye Liu, Weina Zhang, Fengwei Huo*, Bin Liu*

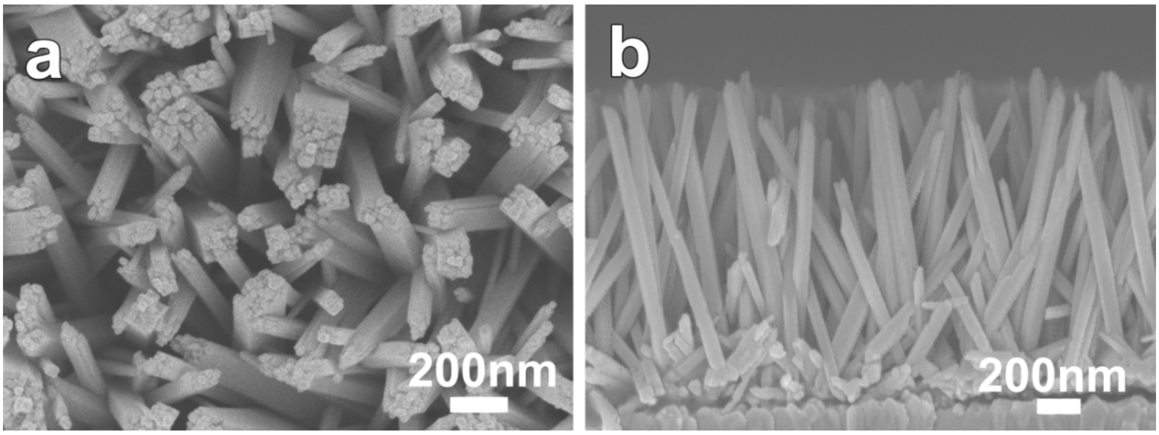

Figure S1. (a) Top view, and (b) cross-sectional view FESEM images of pristine TiO_2 nanowire arrays on FTO.

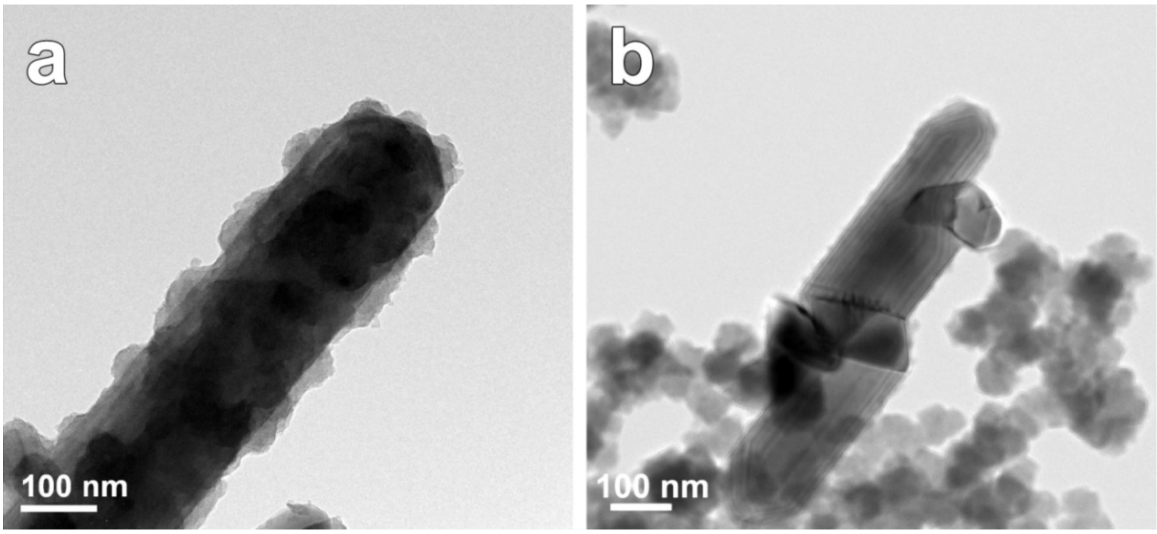

Figure S2. TEM images of MIL-125(NH_2) grown on TiO_2 nanowires (a) with and (b) without ligand surface modification.

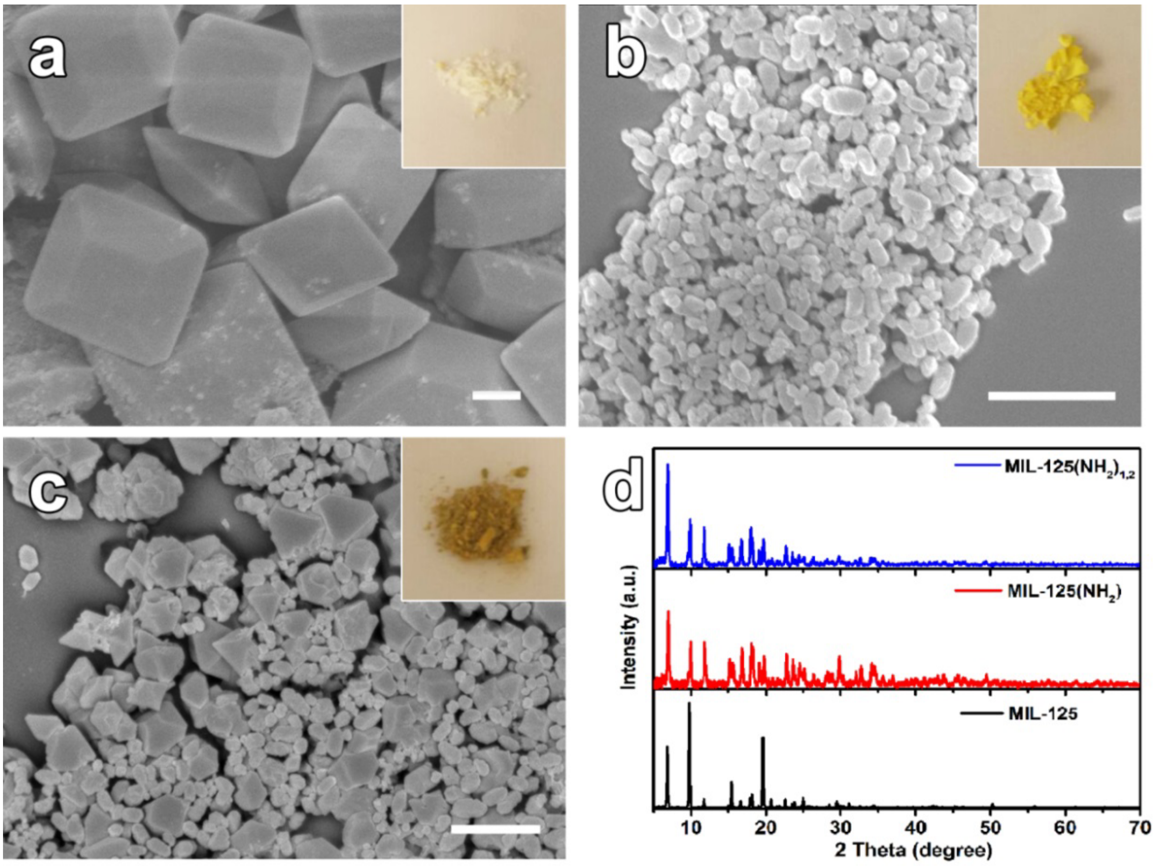

Figure S3. FESEM images of (a) MIL-125, (b) MIL-125(NH_2), and (c) MIL-125(NH_2)$_{1,2}$. (d) XRD patterns of the Ti-based MOFs. Insets show the digital photographs of the as-prepared Ti-based MOFs. Scale bars: 1μm.

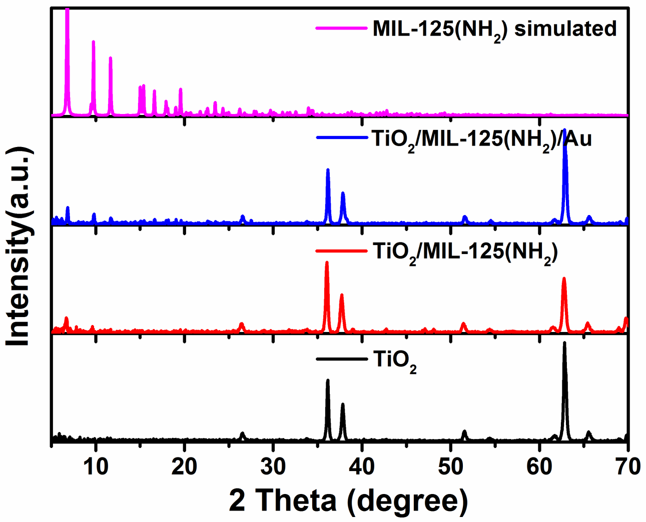

Figure S4. XRD patterns of TiO_2 and Ti-based MOF sensitized TiO_2 nanowires.

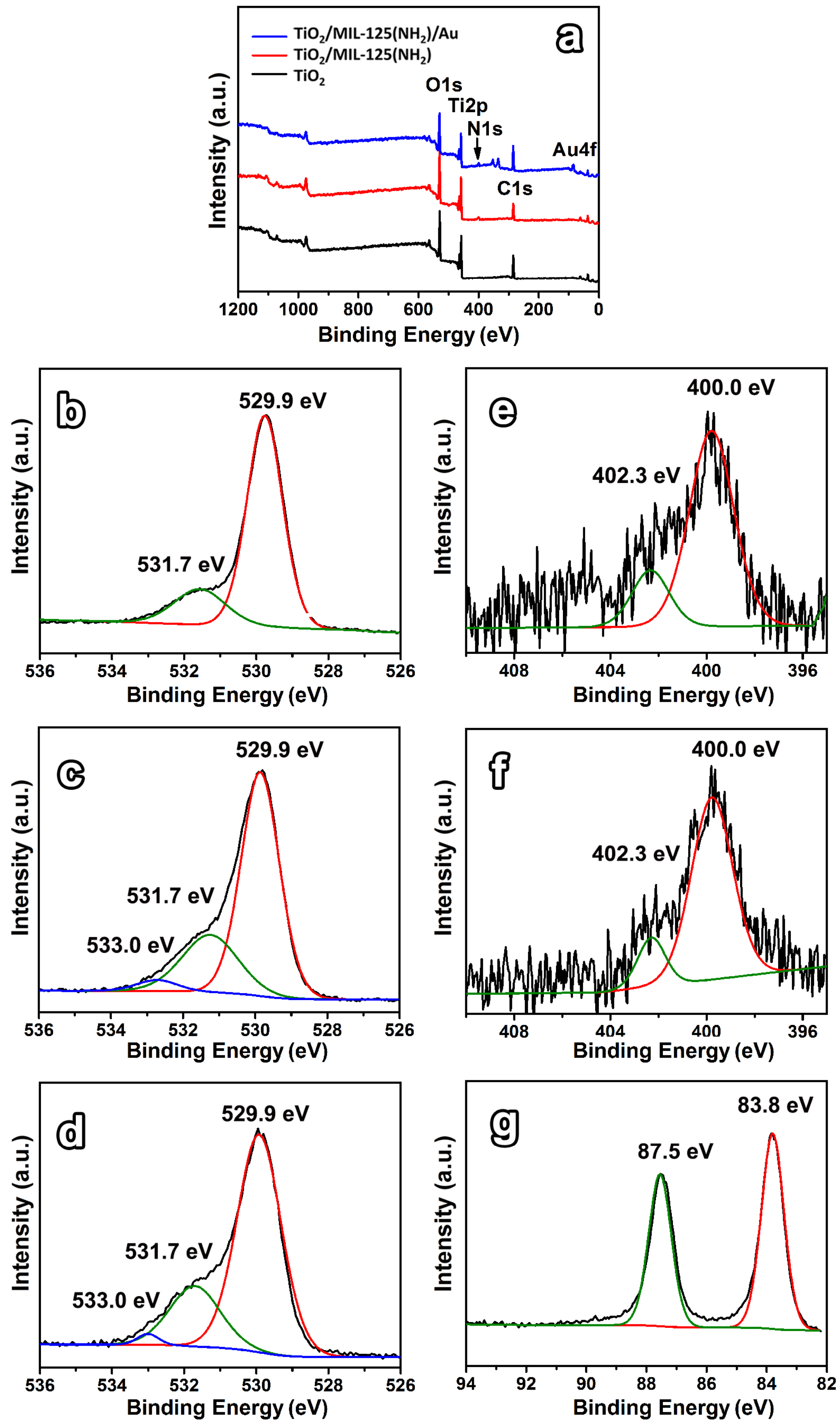

Figure S5. XPS spectra of pristine and sensitized TiO_2 photoanodes. (a) Wide-scan spectra, (b) O1s for TiO_2, (c) O1s for TiO_2/MIL-125(NH_2), (d) O1s for TiO_2/MIL-125(NH_2)/Au, (e) N1s for TiO_2/MIL-125(NH_2), (f) N1s for TiO_2/MIL-125(NH_2)/Au, and (g) Au 4f for TiO_2/MIL-125(NH_2)/Au.

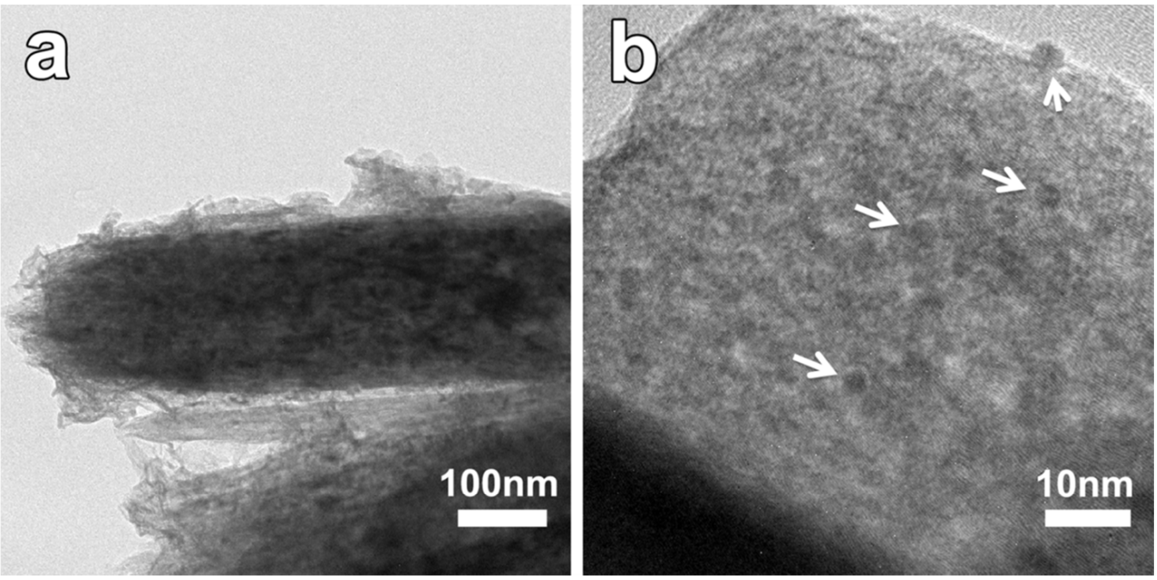

Figure S6. (a) & (b) TEM images of TiO_2/MIL-125(NH_2)/Au.

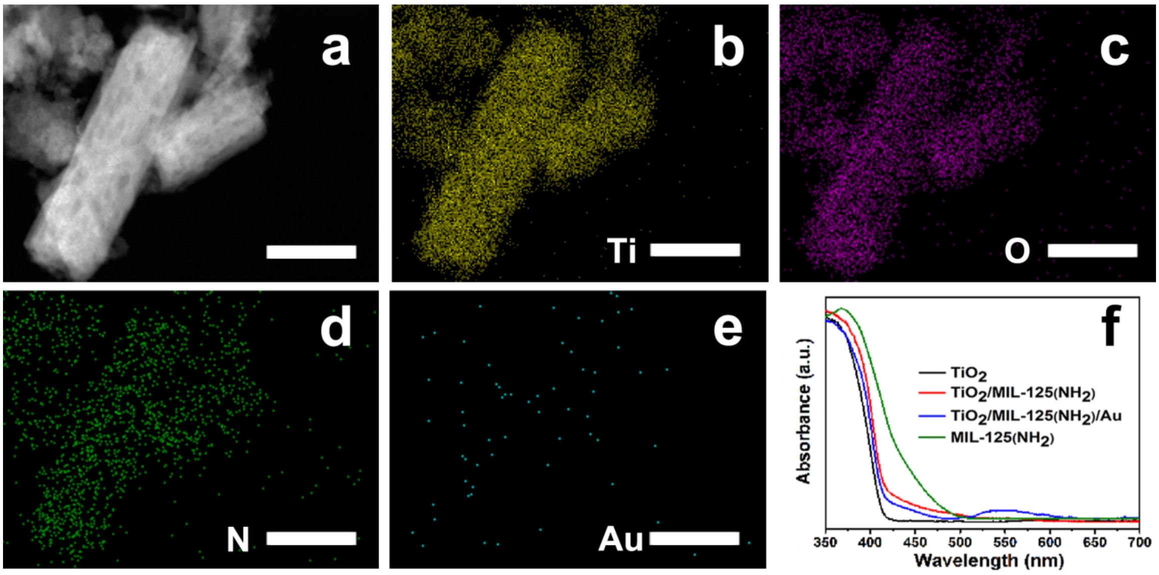

Figure S7. (a) Dark field-STEM image, (b) to (e) corresponding EDS mapping spectra (b: Ti, c: O, d: N, e: Au, scale bars: 200 nm) of TiO_2/MIL-125(NH_2)/Au, and (f) UV-vis diffuse reflectance spectra.

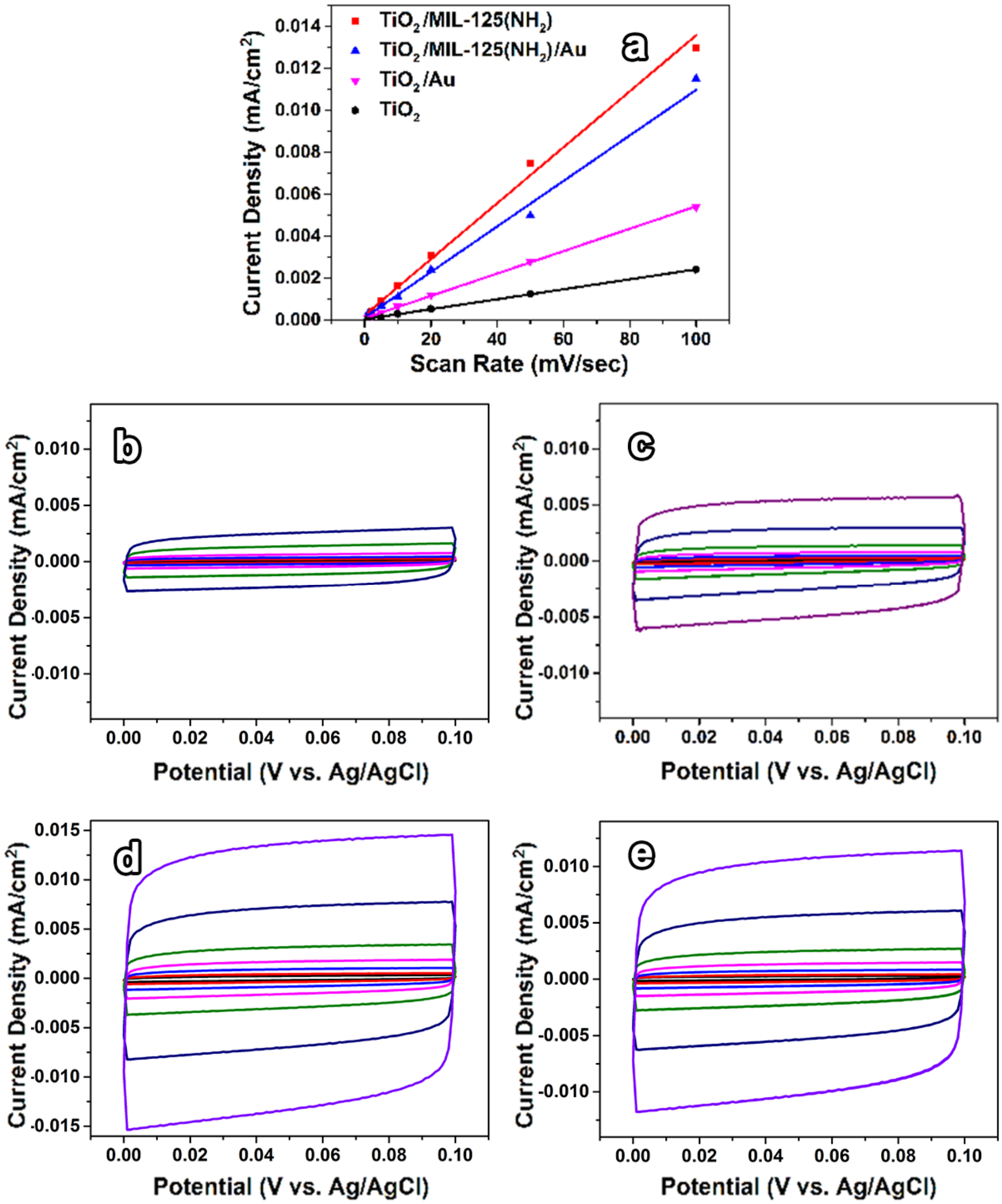

Figure S8. (a) Capacitive current, $\Delta i = (i_{cathodic} - i_{anodic})/2$, as a function of scan rate. The CV scans of (b) TiO_2, (c) TiO_2/Au, (d) TiO_2/MIL-125(NH_2), and (e) TiO_2/MIL-125(NH_2)/Au recorded at scan rates of 1, 2, 5, 10, 20, 50 and 100 mV/s.

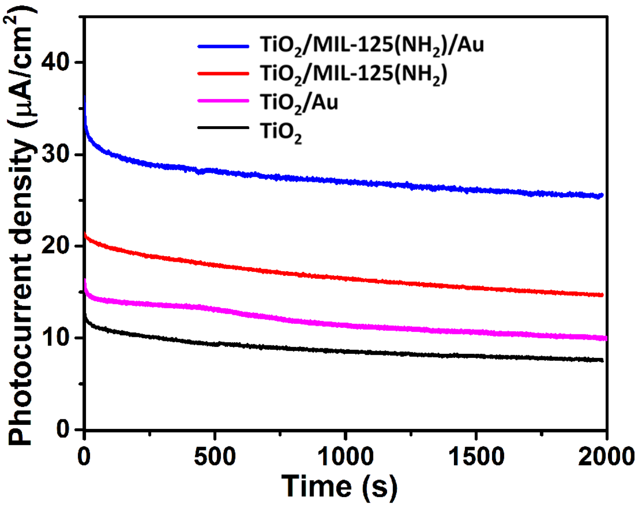

Figure S9. Stability tests of TiO_2 and sensitized TiO_2 nanowire photoanodes: *J*-t curves upon visible light illumination (λ > 420 nm) at 0.75 V vs. RHE.
